# Maternal antagonism of Glp1 reverses the adverse outcomes of sleeve gastrectomy on mouse offspring

**DOI:** 10.1172/jci.insight.156424

**Published:** 2022-04-08

**Authors:** Liron Hefetz, Rachel Ben-Haroush Schyr, Michael Bergel, Yhara Arad, Doron Kleiman, Hadar Israeli, Itia Samuel, Shira Azulai, Arnon Haran, Yovel Levy, Dana Sender, Amihai Rottenstreich, Danny Ben-Zvi

**Affiliations:** 1 Department of Developmental Biology and Cancer Research, Institute of Medical Research Israel-Canada, Hebrew University-Hadassah Medical School, Jerusalem, Israel.; 2 Department of Military Medicine and Tzameret, Faculty of Medicine, Hebrew University of Jerusalem, Jerusalem, Israel, and Medical Corps, Israel Defense Forces, Israel.; 3 Department of Obstetrics and Gynecology and; 4 Faculty of Medicine, Hadassah-Hebrew University Medical Center, Jerusalem, Israel.

**Keywords:** Metabolism, Diabetes, Embryonic development, Obesity

## Abstract

Mothers that underwent bariatric surgery are at higher risk for delivering a small-for-gestational age (SGA) infant. This phenomenon is attributed to malabsorption and rapid weight loss following surgery. We compared pregnancy outcomes in lean mice that underwent sham surgery or sleeve gastrectomy (SG). SG led to a reduction in glucose levels and an increase in postprandial levels of glucagon-like peptide 1 (Glp1) without affecting mice weight during pregnancy. Pups of SG-operated mice (SG pups) were born SGA. The placenta and pancreas of the pups were not affected by SG, although a high-fat diet caused hepatic steatosis and glucose intolerance in male SG pups. Treatment with a Glp1 receptor antagonist during pregnancy normalized the birth weight of SG pups and diminished the adverse response to a high-fat diet without affecting glucose levels of pregnant mice. The antagonist did not affect the birth weight of pups of sham-operated mice. Our findings link elevated Glp1 signaling, rather than weight loss, to the increased prevalence of SGA births following bariatric surgery with metabolic consequences for the offspring. The long-term effects of bariatric surgery on the metabolic health of offspring of patients require further investigation.

## Graphical abstract

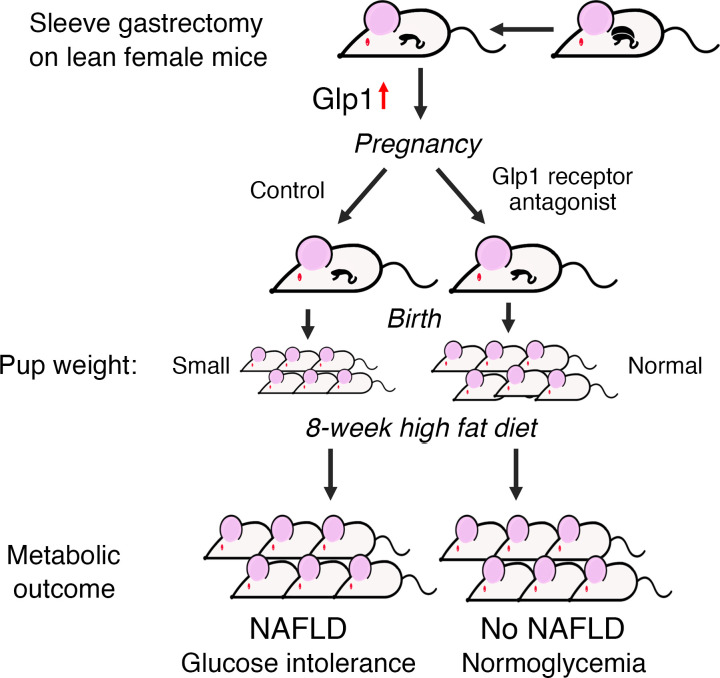

## Introduction

Intrauterine embryo growth is affected by maternal nutrition, systemic metabolism, and endocrine signals (1–5). Malnutrition, in particular a diet poor in protein or severe caloric restriction, can cause intrauterine growth retardation (IUGR) leading to infants that are born small-for-gestational age (SGA), i.e., their birth weight is within the lowest decile of the population. Infants that are SGA are at higher risk of developing obesity and metabolic diseases later in life (2, 3, 5–9). Clinical studies and studies in animal models of IUGR showed that the intrauterine environment can adversely affect pancreatic development, reduce β cell mass and function (10, 11), and cause abnormalities in placental development and its ability to support embryonic growth (1, 12–14). In contrast, gestational obesity and diabetes can cause embryonic hyperinsulinemia and lead to infants that are large-for-gestational age (LGA), which is associated with a higher risk of obesity and metabolic disorders in adulthood (4, 15, 16).

Bariatric surgery usually leads to weight loss and improvement in glycemic control in patients with obesity, particularly in women of reproductive age (17). Bariatric surgery reduces the risk of gestational diabetes, LGA births, and several other risks associated with pregnancy in obese women. Yet it was found that SGA births are more common in women who underwent bariatric surgery (18–23). This outcome can be explained by the substantial weight loss and malabsorption that can follow bariatric surgery (6, 18, 20, 23–25). However, SGA was also recorded following sleeve gastrectomy (SG), which has a smaller malabsorptive component than gastric bypass bariatric surgery. Moreover, SGA is prevalent years after surgery and after the early phase of rapid weight loss following surgery (21, 25–30). The long-term effect of maternal bariatric surgery on offspring is not clear, although some studies report that obesity is less common in offspring of patients who underwent bariatric surgery, compared with their siblings born before surgery (31–35). Mechanistic studies in patients demonstrated that maternal bariatric surgery affected the DNA methylation patterns in the blood of offspring (36–38).

Bariatric surgery results in physiological changes that are not directly related to weight loss. For example, bariatric surgery accentuates postprandial secretion of the incretin glucagon-like peptide 1 (Glp1) and insulin (24). Postprandial hyperinsulinemia leads to a sharp reduction in plasma glucose levels and, in some cases, to postprandial hypoglycemia, which was also recorded in pregnant women who underwent bariatric surgery (39, 40). The surgery also affects the dynamics and levels of ghrelin, bile acids in the plasma, the composition of the gut microbiome, and numerous other factors (41–45) that may impact embryonic growth and development (46–48).

The clinical observations of SGA births following bariatric surgery prompted us to hypothesize that bariatric surgery affects embryonic development and long-term offspring metabolic health independent of maternal weight loss (49–52). To test this hypothesis, we performed SG or sham surgery in lean female mice and mated them with lean male mice. We show that SGA is prevalent in pups born to SG-operated mice (SG pups), even though the mice in both groups reached the same weight at term and had the same number of pups per litter. SG did not affect the development of the placenta or the endocrine pancreas nor had a significant effect on embryonic insulin levels. Male pups born to SG-operated female mice developed hepatic steatosis and glucose intolerance when challenged with a high-fat high-sucrose diet. Treatment with the Glp1 receptor antagonist Exendin 9-39 during pregnancy of mice that had SG normalized the embryo weight and reduced the severity of the metabolic phenotype of male offspring.

## Results

### SG in lean female mice lowers weight and plasma glucose levels.

**Figure 1:**
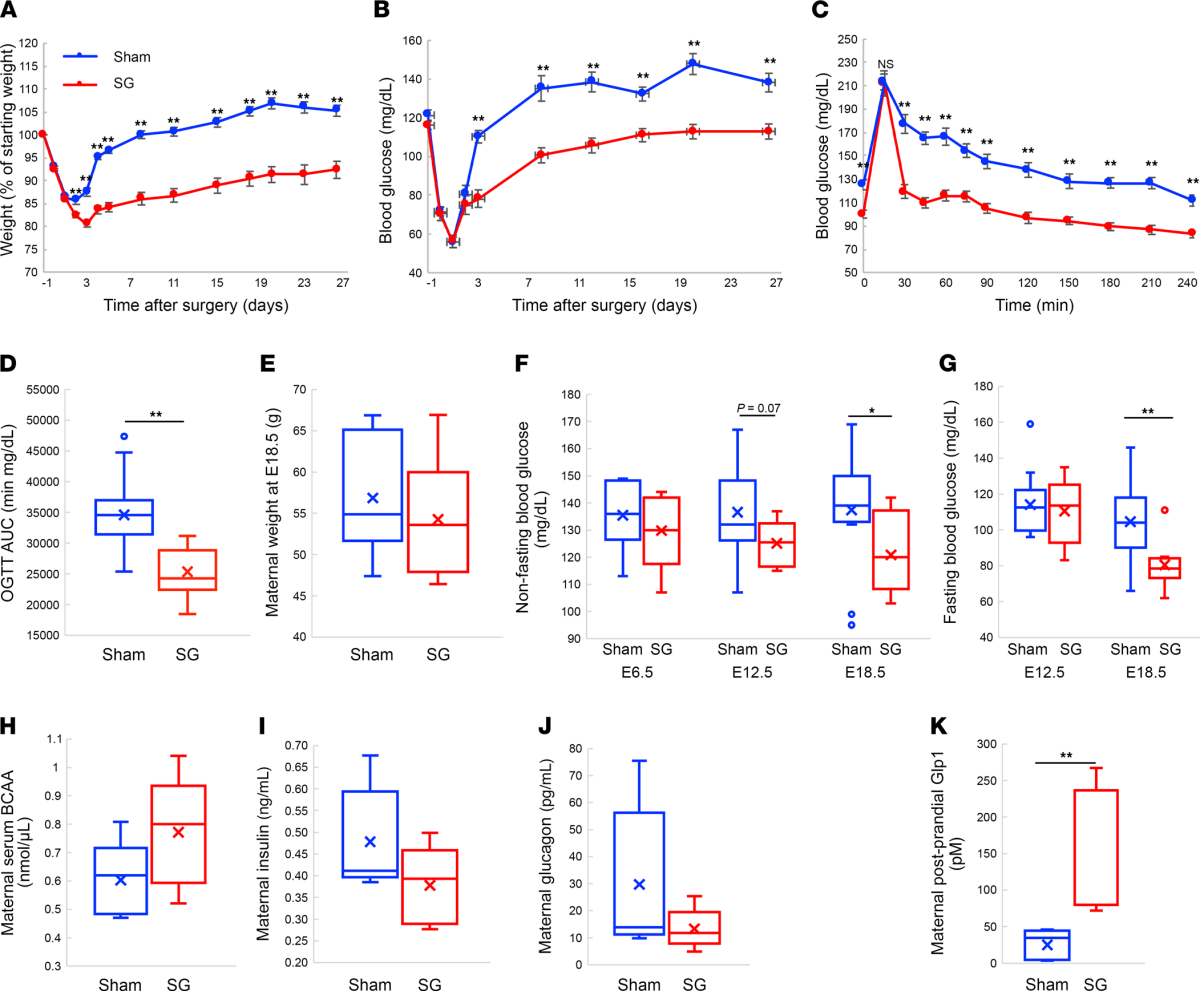
The effects of SG or sham surgery on female mice before and during pregnancy. (**A** and **B**) Mice weight and blood glucose measured at 7 am in the month after SG. (**C** and **D**) Glucose levels and AUC following an oral glucose tolerance test. (**E**) Mice weight at E18.5. (**F** and **G**) Nonfasting and fasting blood glucose during pregnancy. (**H**–**J**) Fasting levels of BCAA, insulin, and glucagon at E18.5. (**K**) Glp1 levels 10 minutes after gavage at E18.5. Blue: sham-operated mice; red: SG-operated mice. In **A**–**D**, sham-operated mice *n* = 28, SG-operated mice *n* = 25; in **E**–**G**, sham-operated mice *n* = 11, SG-operated mice *n* = 10; in **H**–**J**, sham-operated mice *n* = 5, SG-operated mice *n* = 5; in **K**, sham-operated mice *n* = 7, SG-operated mice *n* = 4. *P < 0.05, **P < 0.01 by 2-way continuous measure ANOVA for **A**–**C**, by 2-tailed Student’s *t* test for **D**–**I**; and by Mann-Whitney nonparametric *U* test for **J** and **K**.

Eight-week old lean female mice were fed ad libitum with normal chow underwent SG or sham surgery. The mice continued with a normal chow diet after surgery and were monitored for the next month. SG-operated dams remained 13% leaner than sham-operated mice (Figure 1A and Supplemental Figure 1A; supplemental material available online with this article; https://doi.org/10.1172/jci.insight.156424DS1). Both groups were normoglycemic, but SG-operated mice had lower morning nonfasting blood glucose (Figure 1B). An oral glucose tolerance test (OGTT) performed 4 weeks after surgery showed lower fasting blood glucose levels and lower glucose levels at all time points, except a higher glucose level 15 minutes after gavage, and a reduction in the glucose AUC (Figure 1, C and D).

### Pregnant mice that underwent SG have high postprandial secretion of Glp1.

A month after surgery, SG-operated mice gained weight at the same rate as sham-operated mice. The mice were then mated with nonoperated lean male mice 1 month after surgery. Both SG- and sham-operated mice were fully fertile and by E18.5, SG- and sham-operated pregnant mice had similar weights (Figure 1E and Supplemental Figure 1B). Pregnant SG-operated mice had lower glycemia at E18.5 but not at E6.5 or E12.5 (Figure 1, F and G). There was no difference in the maternal 6-hour fasting plasma levels of branched-chain amino acids (BCAA) (Figure 1H), suggesting that SG does not cause drastic protein malabsorption. There was no difference in insulin or glucagon levels at E18.5 (Figure 1, I and J). The levels of Glp1 were significantly higher 10 minutes after gavage of a liquid diet in SG-operated mice at E18.5 (Figure 1K).

### SG does not affect the placenta at E18.5.

**Figure 2:**
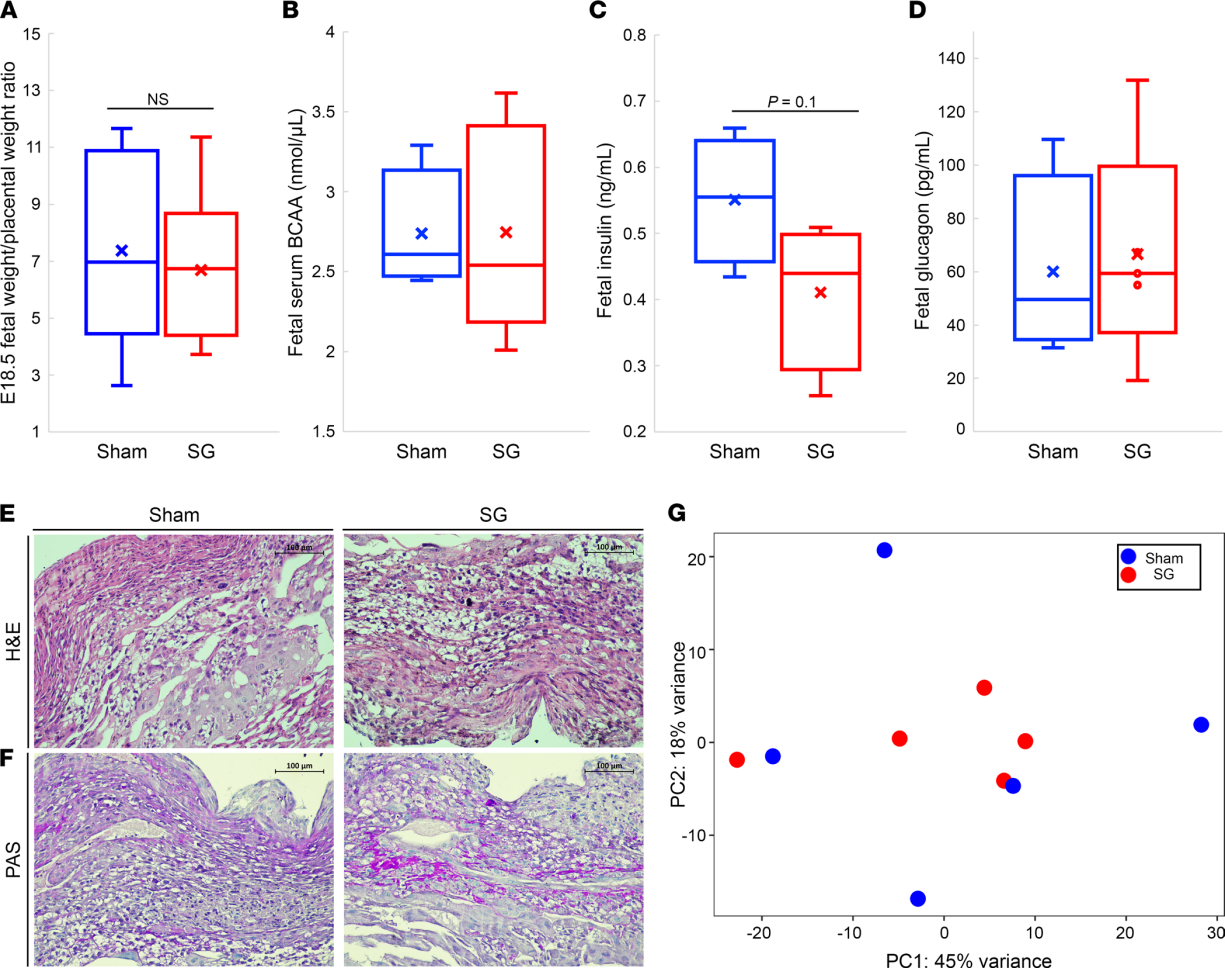
The effects of SG and sham surgery on the placenta. (**A**) Fetal weight to placental weight ratio in SG- and sham-operated mice. (**B**–**D**) Fetal plasma levels of BCAA, insulin, and glucagon. (**E** and **F**) Representative H&E and PAS staining of a placenta from a sham pup (left) or SG pup (right). (**G**) PCA analysis of RNA-Seq of placentas from a pup from each litter. Blue: sham pups and placentas, red: SG pups and placentas. *P* value determined by 2-tailed Student’s *t* test for **A**–**D**. In **A**, sham-operated mice *n* = 13, SG-operated mice *n* = 9; **B**–**D**, sham-operated mice *n* = 4, SG-operated mice *n* = 5. Scale bar: 100 µm.

We extracted fetuses from 5 SG- and 5 sham-operated mice at E18.5. Fetuses of SG- and sham-operated mice had the same fetal weight to placenta weight ratio (Figure 2A) in support of normal placental function. Embryos from both groups had comparable 6-hour–fasted plasma levels of BCAA, insulin, and glucagon (Figure 2, B–D). There was no difference in placental histology in either group (Figure 2, E and F; and Supplemental Figure 2, A and B) (50, 52). mRNA-Seq of whole placentas showed that only a handful of genes were differentially expressed between placentas of SG- and sham-operated mice (Supplemental Figure 2C) with no enrichment of any biological pathway. Unsupervised clustering of gene expression did not separate the 2 groups, and a principal component analysis has shown that surgery type did not affect gene expression (Figure 2G). In particular, there was no difference in the transcript levels of glucose and amino acid transporters, leptin receptor, and Glp1 receptor (49, 50, 52, 53) (Supplemental Figure 2D).

### SG pups have lower birth weight and weigh less until weaning.

**Figure 3:**
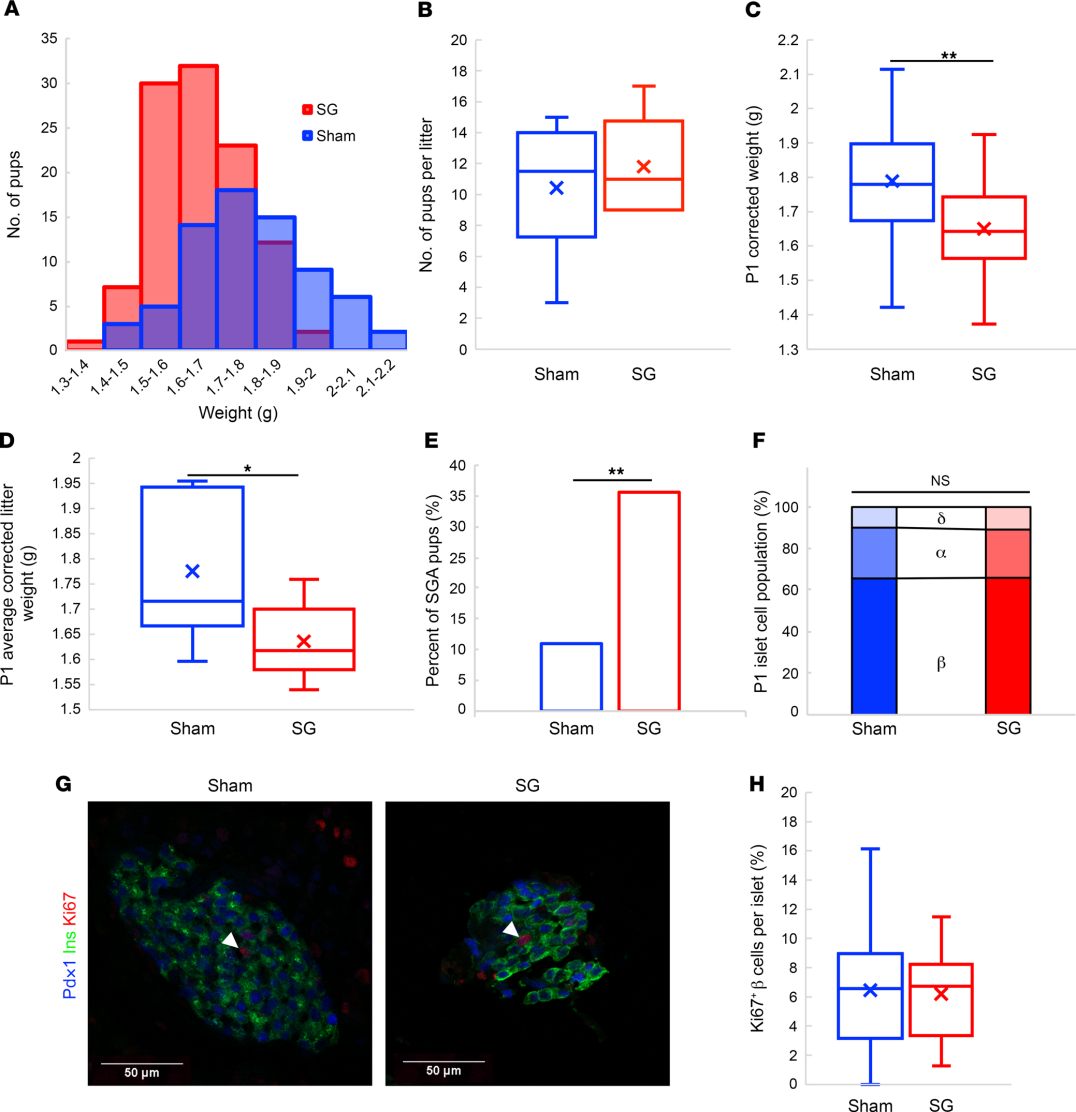
Offspring of SG-operated mice are born SGA. (**A**) Distribution of the weights of P1 pups of mice that underwent sham or SG surgery. (**B**) Litter size of mice that underwent sham or SG surgery. (**C**) P1 weight corrected for litter size in offspring of sham- or SG-operated mice. (**D**) Average corrected weight per litter in mice that underwent sham or SG surgery. (**E**) Fraction of SGA pups (white) in offspring of sham or SG-operated mice. (**F**) Fractions of α, β, and δ cells in the pancreata of P1 mice. (**G** and **H**) Representative image and quantification of Ki67 positive cells in pancreata of P1 mice. Blue: sham pups; red: SG pups. *P < 0.05, **P < 0.01 by 2-tailed Student’s *t* test for **B**–**D** and **H**; and by χ^2^ test for **E** and **F**. In **B**, sham pups *n* = 16, SG pups *n* = 10; in **A** and **C**, sham pups *n* = 72, SG pups *n* = 107; in **D**, sham pups *n* = 7, SG pups *n* = 10; in **E**, sham pups *n* = 26, SG pups *n* = 19. At least 8 islets/P1 pancreas were analyzed, and at least 1 pup per litter was analyzed in **F** and **H**.

SG pups had a lower weight at P1 than pups born to sham-operated dams (sham pups) (Figure 3A). There was no difference in the litter size between both groups (Figure 3B). Litter size was negatively correlated with birth weight (Supplemental Figure 3A), and, therefore, we adjusted pup weight according to litter size (Supplemental Figure 3B). Mean adjusted SG pup weight was smaller by approximately 7% than sham pup weight when considering each pup separately (Figure 3C) and when grouping siblings (Figure 3D). SGA and LGA were defined by the lower and upper 10^th^ percentiles in the corrected weights of the sham pups accordingly. 35% of the SG pups were SGA (Figure 3E), and 0% were LGA (Supplemental Figure 3C).

Each mouse was allowed to nurture 5–6 pups of its litter, and the endocrine pancreata of the remaining pups were analyzed histologically. We found no difference in the distribution of the islet area, in the proportions of α,β, or δ cells in islets at P1. Ucn3 fluorescence, which marks β cell maturation, was equally low in all islets. Neither was there a difference in the fraction of proliferating β cells at this stage (Figure 3, F–H; and Supplemental Figure 3, D–G).

**Figure 4:**
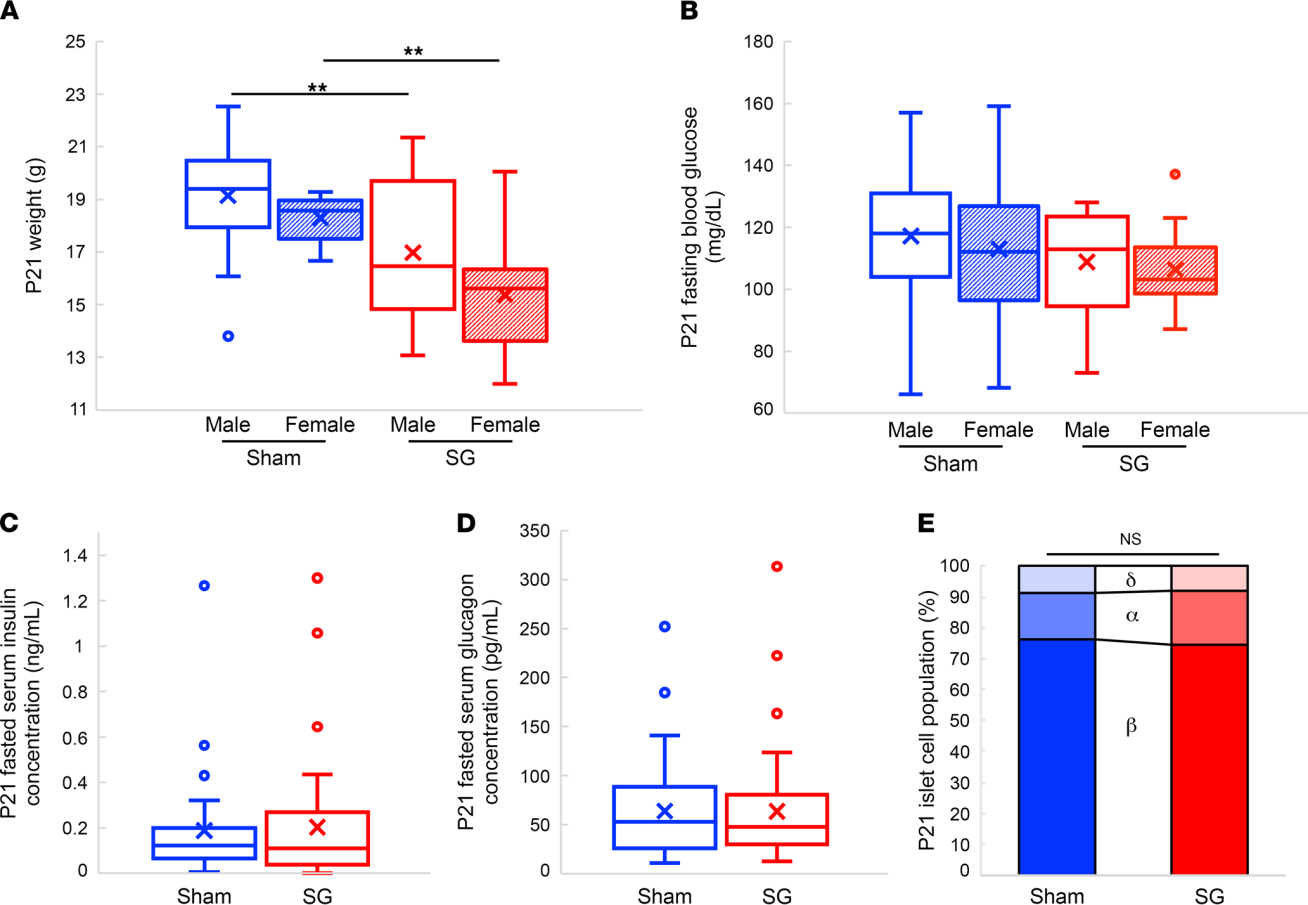
Offspring of SG-operated mice remain smaller than offspring of sham-operated mice until weaning. (**A** and **B**) Weight and fasting blood glucose at P21 of male and female offspring of sham- and SG-operated mice. (**C** and **D**) Fasting level of insulin and glucagonat P21 in offspring of sham- and SG-operated mice. (**E**) Fractions of α, β, and δ cells in the pancreata of P21 mice. Blue: offspring of sham-operated mice, red: offspring of SG-operated mice. **P < 0.01 by 2-way ANOVA with Tukey HSD post hoc test in **A** and **B**; by 2-tailed Student’s *t* test in **C** and **D**; and by χ^2^ test in **E**. In **A** and **B**, sham-operated male mice *n* = 23, sham-operated female mice *n* = 16, SG-operated male mice *n* = 26, SG-operated female mice *n* = 25; in **C**, sham-operated mice *n* = 38, SG-operated mice *n* = 38; in **D**, sham-operated mice *n* = 40, SG-operated mice *n* = 46; and in **E**, cumulative of pancreata of 10 mice each from sham- and SG-operated mice.

At P21, male SG pups weighed 11% less than male sham pups, and female SG pups weighed 16% less than female sham pups (Figure 4A and Supplemental Figure 4A). There was no difference in fasting blood glucose, insulin, or glucagon levels (Figure 4, B–D). There was no difference in the proportions of islet cell composition between SG pups and sham pups or in islet size distributions (Figure 4E and Supplemental Figure 4, B and C).

### Inhibition of Glp1 receptor during pregnancy reverses the effects of SG on offspring birth weight.

**Figure 5:**
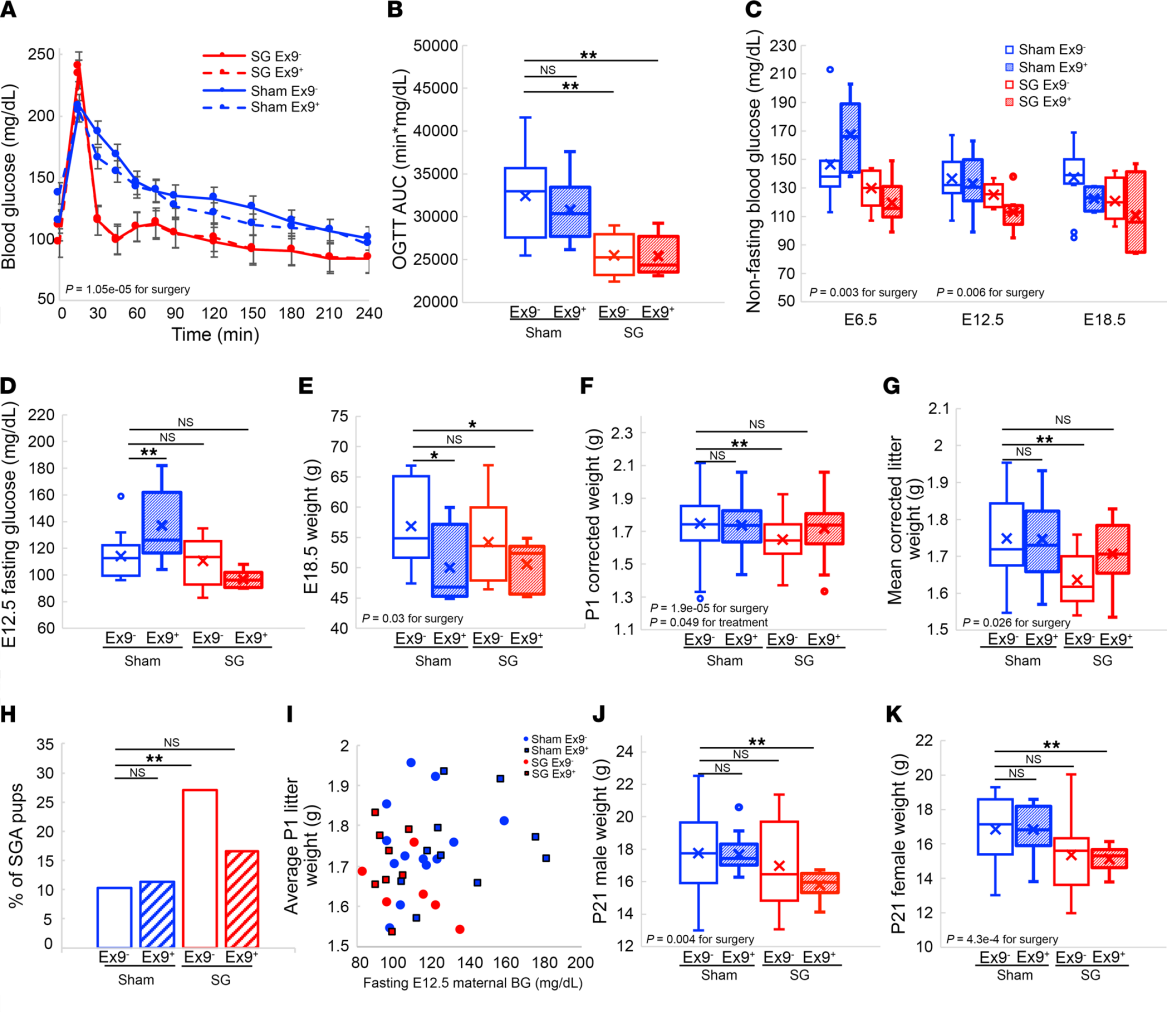
Treatment with Exendin 9-39 normalizes the effects of SG on embryo size without affecting maternal glycemia. (**A** and **B**) Oral glucose tolerance test and AUC in pregnant mice (E12.5) that had sham or SG surgery and were treated or not treated with Exendin 9-39 during pregnancy. (**C** and **D**) Nonfasting and fasting glucose levels of the 4 groups of mice. (**E**) Weight at E18.5 of the 4 experimental groups. (**F** and **G**) Corrected weight of P1 pups and the average weight of litters in the 4 experimental groups. (**H**) Fraction of SGA pups in the 4 experimental groups. (**I**) Scatter plot of birth weight as a function of fasting glycemia in E12.5. (**J** and **K**) Weight of male and female offspring of the 4 experimental groups. Blue: sham-operated mice or offspring of sham-operated mice untreated with Exendin 9-39 during pregnancy (full) or treated with Exendin 9-39 (dashed); Red: SG-operated mice or offspring of SG-operated mice untreated with Exendin 9-39 during pregnancy (full) or treated with Exendin 9-39 (dashed). *P < 0.05, **P < 0.01 by 2-way ANOVA with Tukey HSD post hoc test in **B**–**J** or by 3-way continuous measures ANOVA in **A**. In **A**–**D**, sham Ex9^–^ *n* = 14, sham Ex9^+^ *n* = 10, SG Ex9^–^ *n* = 6, SG Ex9^+^ *n* = 9; in **E**, sham Ex9^–^ *n* = 14, sham Ex9^+^ *n* = 10, SG Ex9^–^ *n* = 6, SG Ex9^+^ *n* = 9; in **F**, sham Ex9^–^ *n* = 155, sham Ex9^+^ *n* = 98, SG Ex9^–^ *n* =107, SG Ex9^+^ *n* = 85; in **G**, sham Ex9^–^ *n* = 16, sham Ex9^+^ *n* = 10, SG Ex9^–^ *n* =10, SG Ex9^+^ *n* = 8; in **I**, sham Ex9^–^ *n* = 13, sham Ex9^+^ *n* = 10, SG Ex9^–^ *n* = 6, SG Ex9^+^ *n* = 9; in **J**, sham Ex9^–^ *n* = 16, sham Ex9^+^ *n* = 16, SG Ex9^–^ *n* = 25, SG Ex9^+^ *n* = 11.

Since postprandial Glp1 levels were markedly increased in SG-operated mice during pregnancy, we postulated that inhibition of Glp1 signaling during pregnancy by the Glp1 receptor antagonist Exendin 9-39 could reverse the effects of SG on pup weight. Exendin 9-39 treatment increased fasting plasma glucose levels and attenuated glucose clearance in an oral glucose tolerance test in virgin, nonoperated female mice (Supplemental Figure 5, A and B). However, the same dose of Exendin 9-39 did not affect fasting glucose levels, response to an oral challenge, or nonfasting glucose levels at the end of pregnancy of both sham- and SG-operated pregnant mice (Figure 5, A–D). Exendin 9-39 led to mild weight loss during pregnancy (Figure 5E).

SG pups born to Exendin 9-39–treated dams had the same weight as untreated sham pups, yet Exendin 9-39 treatment did not affect the weight of sham pups (Figure 5, F and G). Accordingly, SGA births of SG pups of Exendin 9-39–treated dams was 16%, and 11% in sham pups of Exendin 9-39–treated dams (Figure 5H). Exendin 9-39 did not increase the fraction of LGA births in sham-operated mice, and induced LGA in 6% of the SG-operated mice (Supplemental Figure 5C). There was no correlation between maternal glycemia and birth weight of pups and, in particular, Exendin 9-39–treated SG-operated mice had low glucose during pregnancy and normal pup weight (Figure 5I). At P21, male and female SG pups weighed less than sham pups regardless of treatment (Figure 5, J and K).

### Long term effect of maternal SG and treatment with Exendin 9-39.

Female offspring of mice treated with Exendin 9-39 were heavier at the age of 8 weeks, while surgery type did not affect weight (Supplemental Figure 6A). The female mice were then mated with lean male mice. Offspring of Exendin 9-39–treated mice maintained higher weight throughout pregnancy (Supplemental Figure 6A). All groups had similar nonfasting blood glucose levels before and during pregnancy (Supplemental Figure 6B). Surgery type or treatment had no significant effect on the weight of the pups (Supplemental Figure 6C).

**Figure 6:**
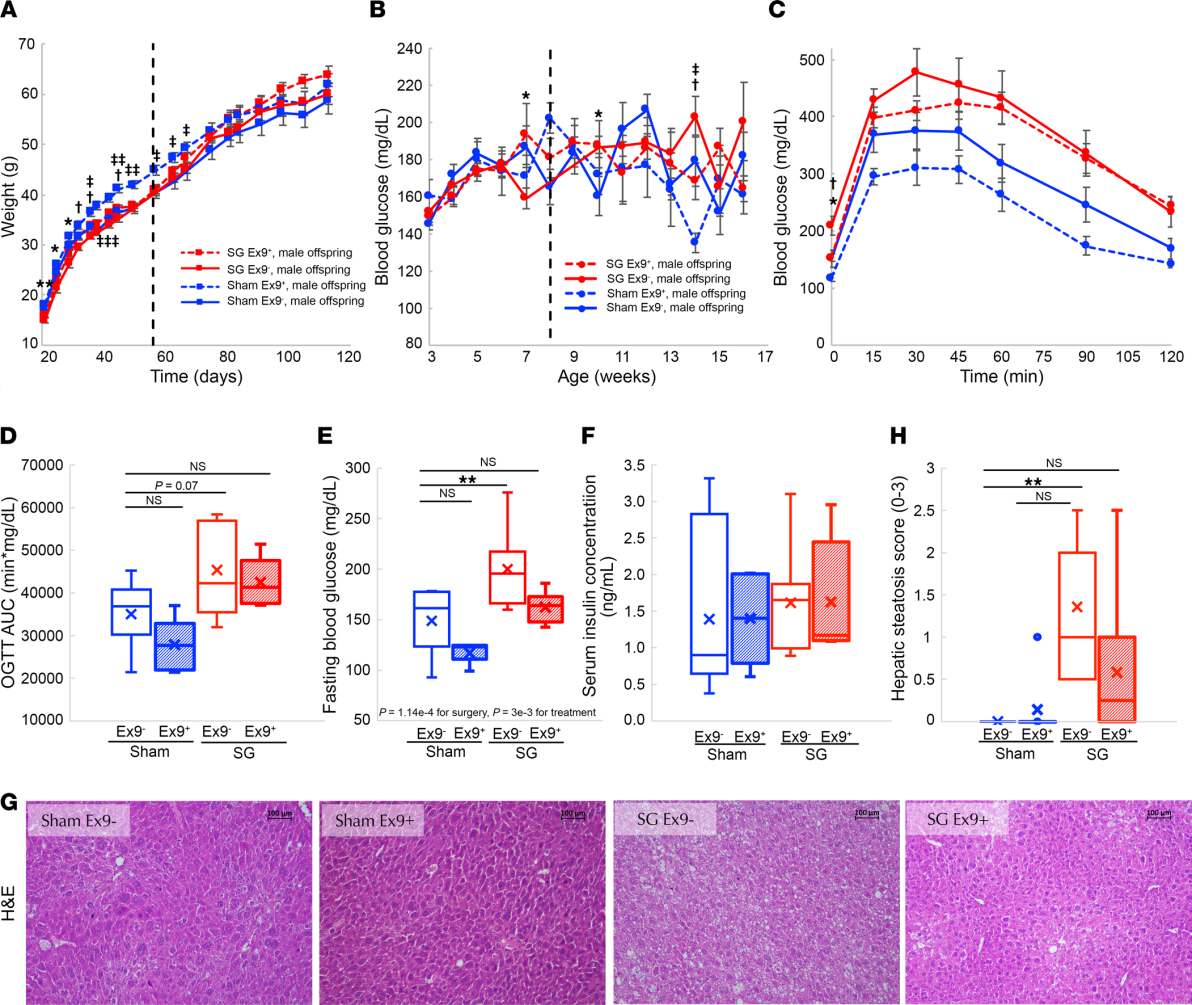
The long-term effects of SG on male offspring are reversed by maternal treatment with Exendin 9-39. (**A** and **B**) Weight and nonfasting glucose levels of male offspring of sham- or SG-operated mice treated or untreated with Exendin 9-39 during pregnancy. The dashed black line denotes a switch from normal chow to a high-fat high-sucrose diet. (**C**–**F**) Oral glucose tolerance test, AUC, fasting glucose, and insulin levels at week 15 of the 4 experimental groups. (**G**) Representative H&E staining of the liver of the 4 experimental groups. (**H**) Histological grade of hepatic steatosis in the 4 experimental groups. Blue: male offspring of sham-operated mice untreated with Exendin 9-39 during pregnancy (full) or treated with Exendin 9-39 (dashed); Red: male offspring of sham-operated mice untreated with Exendin 9-39 during pregnancy (full) or treated with Exendin 9-39 (dashed). *P < 0.05, **P < 0.01 by 2-way ANOVA with Tukey HSD post hoc test in D, F, H, and I or by 3-way continuous measures ANOVA in **A**–**C** and **E**. All panels sham Ex9^–^ *n* = 7, sham Ex9^+^ *n* = 7, SG Ex9^–^ *n* = 7, SG Ex9^+^ *n* = 6. *P < 0.05, **P < 0.01 for the comparison between sham-Ex9^–^ and SG-Ex9-. ^†^*P* < 0.05, ^††^*P* < 0.01 for the comparison between SG-Ex9^–^ and SG-Ex9+. ^‡^*P* < 0.05, ^‡‡^*P* < 0.01 for the comparison between sham-Ex9^–^ and sham-Ex9+.

Male offspring of SG- and sham-operated dams were fed on normal chow for 8 weeks. At that point, offspring of Exendin 9-39–treated sham-operated mice weighed 10% more than offspring of other groups (Figure 6A). Mice were then provided with a high-fat high-sucrose diet. After 8 weeks, there was no difference in the weights of the experimental groups (Figure 6A).

There was no difference in nonfasting glucose levels throughout the experiment between the groups (Figure 6B). OGTT performed at week 15 revealed that offspring to SG-operated mice had higher fasting glucose and worse response to a glucose challenge compared with offspring of sham-operated mice. Maternal Exendin 9-39 treatment was associated with lower fasting glucose and better response to an OGTT. There was no difference in fasting insulin levels (Figure 6, C–F). Male offspring of SG-operated mice displayed hepatic steatosis. Hepatic steatosis was improved in offspring of Exendin 9-39–treated SG-operated mice and was not observed in the offspring of sham-operated mice (Figure 6, G and H). There was no difference in the plasma levels of glucagon, albumin, hepatic enzymes, or lipids between the experimental groups (Supplemental Figure 7, A–D).

## Discussion

In this study, we show that lean female mice that underwent SG are fertile and give birth to pups that weigh less than pups born to mice that underwent sham surgery. A third of the SG pups were born SGA. We did not identify a developmental defect in the placenta or the endocrine pancreas of the embryos and pups. However, male SG pups developed hepatic steatosis and glucose intolerance upon exposure to a high-fat high-sucrose diet. Treatment during pregnancy with Exendin 9-39, a Glp1 receptor antagonist, reduced the prevalence of SGA and improved glycemic parameters of adult offspring of SG-operated dams.

Caloric restriction and protein deficiency can lead to IUGR and a reduction in β cell mass and function in rodents (5, 6, 10, 11), possibly since fetal islets secrete insulin in response to amino acids (54, 55). SG-operated mice, however, gained weight normally during pregnancy and had the same weight as sham-operated mice at term. The levels of BCAA were similar in the plasma of SG- and sham-operated dams and also in the plasma of their offspring, suggesting no major deficiency in protein digestion, absorption, or transfer of amino acids to the embryos. Correspondingly, there was no histological phenotype in offspring β cell development during the postnatal stages we examined, and the body weight to placental weight ratio was not affected by surgery. SG does not have a major malabsorptive component, especially a short time after surgery, but we cannot exclude a deficiency in micronutrients (56) that might affect pregnancy outcomes (55).

Placental insufficiency, reduction in transport of nutrients across the placenta, or defects in the development of the placenta contribute to fetal growth retardation (1, 2, 12, 13). Previous studies in rodent models described adverse effects of SG on the placenta when the animals were fed a high-fat diet, but these effects were not observed on a normal chow diet, in agreement with the results we presented (50, 52). SG had no effect on the transcriptome or histology of the placenta at E18.5 in our study (57). Current clinical literature now shows an association between a history of bariatric surgery and placental defects, unless major micronutrient deficiency or smoking developed after surgery (20, 58). Together, the results suggest that SG does not cause significant changes in placental development.

Fetal growth is driven in part by fetal insulin in humans and mice (55, 59). While there is strong evidence that maternal hyperglycemia leads to fetal hyperinsulinemia and increased fetal growth (55, 60), it is not established that lower maternal glucose levels, especially within the normal range, can cause fetal hypoinsulinemia and decreased growth (54). As we and others show, fetal glycemia is normally much lower than maternal glycemia (Supplemental Figure 7E) (54). Lower birth weight was associated with hypoglycemia during OGTT in pregnant women who underwent bariatric surgery (39), and SG-operated dams displayed low levels of glucose compared with lean, sham-operated dams in an OGTT during pregnancy. Yet the correlation between maternal glycemia and adjusted embryo weight at P1 in our study was lost once we consider the Exendin 9-39–treated groups (Figure 5I). Low glucose levels after bariatric surgery are associated with an elevation in the levels of several hormones, including Glp1 (40, 61). Indeed, postprandial levels of Glp1 were 2-fold higher in SG-operated mice (Figure 1K). These results raise the hypothesis that Glp1 signaling leads to a reduction in glucose levels and affects fetal size via 2 pathways.

Inhibition of Glp1 signaling normalized embryo weight in SG-operated mice. A simple explanation for this effect of Exendin 9-39 is that the treatment caused maternal weight gain and hyperglycemia, leading to increased fetal growth (55). However, we did not detect any difference in maternal glycemia in Exendin 9-39–treated mice in either surgical group, and Exendin 9-39 treatment was not associated with higher maternal weight at the end of pregnancy. Exendin 9-39 did not affect the weight of sham pups. We conclude that high Glp1 rather than low glucose is a key effector of embryo size after SG in lean mice. Furthermore, the finding that Exendin 9-39 increases pup weight provides evidence against the hypothesis that malabsorption is the main cause for SGA in this model. Offspring of SG-operated mice remained leaner by P21 regardless of Exendin 9-39 treatment but their weight caught up after weaning. Factors such as the composition of milk or maternal microbiome following surgery may also contribute to the postnatal differences between offspring of SG- and sham-operated mice (53, 62, 63).

Semaglutide and Liraglutide are stable Glp1 receptor agonists that are used to treat patients with hyperglycemia. The administration of these drugs is prohibited during pregnancy because animal studies have shown that these agonists reduce embryo size and cause developmental abnormalities (64, 65). These findings support a role for high Glp1 signaling in reducing embryo size. The elevation in Glp1 signaling in SG-operated mice did not cause developmental defects that were reported in Liraglutide- or Semaglutide-treated animals (50–52, 64, 65); neither are such abnormalities common in patients that underwent bariatric surgery (20, 22, 23). One possible explanation is that, while Semaglutide and Liraglutide are stable and can cross the placenta, the placental transport of Glp1 is minor (66–68).

The Glp1 receptor is robustly expressed in β cells, lungs, a subset of neurons and glia in the adult mouse, and several other cell types (69), but lowly expressed in the mouse placenta at E18.5 (Supplemental Figure 2D) in both SG- and sham-operated mice. Glp1 signaling may affect maternal androgen metabolism through its effects on insulin. Androgens have a sex-specific effect on fetal β cell and adrenal development, which can contribute to the sex-specific effect of SG and Exendin 9-39 treatment on the response of offspring to a metabolic challenge (70–73). Overall, we hypothesize that Glp1 signaling exerts its effect on the mother, affecting the fetus indirectly to reduce growth. In line with this hypothesis, postnatal treatment with the Glp1 receptor agonist Exendin 4 in animals that suffered from experimental IUGR reduced the adverse metabolic phenotypes of fetal IUGR in adulthood (10, 14).

Ghrelin, a peptide hormone secreted from the stomach, contributes to fetal growth in late gestational stages. Maternal ghrelin crosses the placenta and affects the embryo directly (47). In SG, most of the stomach is removed, resulting in a sharp reduction in the levels of ghrelin (44), which can contribute to the high prevalence of SGA in pups and infants born to mothers who had SG. In this case, Glp1-signaling inhibition creates an independent, opposite effect to that of the reduction in ghrelin signaling. There are several endocrine, immune, and metabolic regulators that are affected by SG that may also affect embryonic growth and development (42, 49–51, 53, 70–74). Our finding that inhibition of Glp1 signaling during pregnancy affects the weight of SG pups, specifically, points to Glp1 as a major, yet nonexclusive, factor that contributes to the high prevalence of SGA in SG-operated dams. The effects of Glp1 receptor inhibition during pregnancy of SG-operated mice in an obese mouse model require further experiments.

SGA is associated with a greater risk of developing glucose intolerance later in life regardless of etiology (7–9). Long-term data on the metabolic health of offspring of patients that underwent bariatric surgery are scarce and conflicting (31–35). We show here that SGA is prevalent in offspring of lean mice that underwent SG, with no background of maternal obesity, no weight loss at term, and no indication of protein malabsorption. Male offspring of SG-operated dams develop hepatic steatosis, fasting hyperglycemia, and greater insulin resistance upon exposure to a high-fat diet compared with offspring of sham-operated dams. Treatment with the Glp1 receptor antagonist Exendin 9-39 during pregnancy reverses the negative effects of surgery on the metabolic health of male offspring. Exendin 9-39 is now under clinical trials to treat postbariatric hypoglycemia, yet there is still no information on its effects during pregnancy or on offspring (61). Further studies are warranted to identify the mechanism by which Glp1 exerts its effects on fetal growth and development and the safety and efficacy of Exendin 9-39 in this context.

## Methods

### Mice

We used Hsd:ICR (CD-1) outbred mouse model fed with normal chow. In the long-term experiment, male offspring were fed a high-fat high-sucrose diet (Envigo, TD08811).

### Surgery

Female ICR mice that were 8-weeks-old underwent SG surgery as described previously (75). Briefly, a midline incision through the skin and underlying *linea alba* was performed, followed by exposure and mobilization of the stomach. A 12 mm clip was placed horizontally across its greater curvature using a Ligaclip Multiple Clip Applier. The excluded part of the stomach was excised, the abdominal wall was sutured using 6-0 coated vicryl sutures (Ethicon, J551G), and the skin was closed with clips (Autoclip by NBT, FST-12022-09). Sham surgeries included the abdominal incision, exposure and mobilization of the stomach, and closure of the body wall and skin. Each surgery lasted approximately 15–20 minutes. Mice were fasted overnight the day before surgery and during the day of surgery, provided with an Ensure liquid diet for 2 days, and then returned to normal chow. Nearly 90 animals were operated on. The survival rate in SG surgery was approximately 85% in SG and 100% in sham surgery.

### P1 weight calculation

Weight at P1 was corrected to account for the negative association between the number of pups per litter and the average pup weight per litter. We used a linear model using litter size as the only parameter to correct the weight and account for this association. The corrected weight equals the difference between the measured P1 weight and the calculated average weight for the specific litter sized by the linear model, plus the modeled weight for litter size of 11, which was the median litter size. See Supplemental Figure 3, A and B.

Weaning: the number of pups was reduced to 5–6 pups per mother at P1, and pups were weaned at P21.

### Hormone measurement

Plasma hormones were measured following a 6-hour fast between 7 am and 1 pm, using Ultra-Sensitive Mouse Insulin ELISA (Crystal Chem, 90080), Mouse Glucagon ELISA (Crystal Chem, 81518), and Total GLP-1 (ver. 2) (Mesoscale, K150JVC-1).

Glp1 receptor antagonism: Exendin 9-39 amide (Sigma Aldrich, E7269) was injected s.c. daily during pregnancy at a concentration of 25 nmol/kg in 0.9% NaCl, normalized to 10 mL/kg.

### Blood glucose, glucose tolerance tests (GTT), insulin tolerance test (ITT)

Blood glucose was measured with a glucometer Accu-Check by Roche by tail bleeding. Nonfasting blood glucose refers to blood glucose level at 7 am when animals had ad libitum access to food and water throughout the night.

OGTT was performed on mice fasted for 6 hours between 7 am and 1 pm by injecting oral gavage 20% glucose solution in saline and measuring blood glucose at 15, 30, 45, 60, 75, 90, 120, 150, 180, 210, and 240 minutes after injection.

ITT was performed by injecting i.p. 1:1000 insulin/saline solution on mice fasted for 6 hours between 7 am and 1 pm and measuring blood glucose at 15, 30, 45, 60, 90, and 120 minutes after injection. Gavage and injections were normalized to body weight by providing 10 mL/kg solution to mice.

E18.5 C-section and sacrifice

On day 18.5 of pregnancy, pregnant mice were fasted for 6 hours then anesthetized using ketamine/xylazine solution. Following an abdominal preparation with 70% ethanol, a V-shaped skin and fascia were incised, allowing exposure of the uterine horns. Using fine forceps and cotton swabs, the uterus was exposed and uterine vessels were identified. Fetuses were extracted with their placenta, then amniotic membranes were removed and their blood was extracted. Some of the placentas were collected and stored in liquid nitrogen then transferred to storage at –80°C, and some were fixated in 4% formaldehyde overnight for later pathology sections.

### Sacrifice

At the end of each experiment, mice were anesthetized using ketamine as a sedative and xylazine as a muscle relaxant, diluted in 0.9% sodium chloride.

Blood was extracted via terminal bleeding using heparin-coated syringes and 25G needles and transferred to lithium heparin-coated tubes (Greiner, MiniCollect, 450535). Aprotinin (Sigma-Aldrich, A6279), DPP4 inhibitor,and EDTA were added to the blood sample to avoid degradation of glucagon and GLP-1. Blood was then centrifuged at 6,000 RCF for 1.5 minutes. Plasma was then collected and stored in liquid nitrogen until transferred to final storage at –80°C.

### RNA extraction

Total RNA was extracted from whole placentas using TRI reagent (Sigma-Aldrich, T9424) according to the manufacturer’s instructions. A single placenta from each litter was used.

### RNA sequencing and analysis

Raw reads were aligned to the mouse genome and transcriptome (genome version GRCm38, with annotations from Ensembl release 99) using the RNA-Seq Alignment App on Basespace (Illumina), following differential expression analysis using DESeq2. Differentially expressed genes were characterized for each sample (|L2FC| > 1, *P* adjusted value < 0.05) and were used as a query to search for enriched biological processes (Gene ontology BP). RNA-Seq data is available online in GEO accession GSE195998.

### Tissue fixation and processing

P1 pancreata were fixed in 4% paraformaldehyde/PBS (PFA/PBS) for 4 hours, and P21 pancreata were fixed in 4% formaldehyde for 2.5 hours at room temperature. Liver samples were fixed overnight at 4°C in 4% formaldehyde, then washed in PBS and continued fixation in 70% ethanol. E18.5 placentas were fixed for 4 hours in 4% paraformaldehyde and stored in PBS. Sections were paraffin-blocked and counterstained with H&E, periodic acid–Schiff (PAS; Merck 1016460001, 1016470500), and DAB stain.

Later, tissue paraffinization was performed in the Hadassah Ein-Kerem Pathology Department then transferred to paraffin blocks. Tissues were cut into 4 μ slides and deparaffinized using Xylene, decreasing the percentage of alcohol.

### Histology and immunofluorescence stain

Antigen retrieval was performed in citrate buffer (pH 6.0) in a pressure cooker. Tissues were blocked in CAS block (Thermo Fisher Scientific) and incubated overnight with primary antibodies diluted in CAS block (Table 1):

Slides were washed 3 times in PBST, incubated overnight with secondary antibodies, and diluted in PBS/BSA 1% (Table 2):

Slides were then washed once in PBS and closed with cover glass using ImmuGio mounting medium (DiaSorin, 2505-5). Relevant tissue samples were incubated with DAPI nuclear stain (Sigma-Aldrich, D9564; diluted 1:100 in double distilled water [DDW]) for 5 minutes, then washed 5 minutes in DDW before closing. Pictures were taken with Nikon confocal microscope, and the software NIS-Elements. For UCN3 fluorescence analysis, the same microscopy and NIS software settings were used per experiment, and fluorescence measurements were taken using ImageJ (NIH) (RGB measurement Plugin).

### H&E stain

#### Tissues were deparaffinized and stained.

5 minutes incubation with freshly filtered Mayer’s Hematoxylin solution (Sigma, MHS32) and wash under running tap water; Dip in 1% acid alcohol (1:100 of 37% HCl solution/80% ethanol) and wash under running water; Dip in 95% ethanol and 1 minute incubation with alcoholic Eosin Y with phloxin (Sigma, HT110332); Dehydration from 95% ethanol to 100% ethanol and Xylene. Dried slides were sealed with Entellan mounting medium (Mercury, 1079600600)

#### Alcian blue-PAS stain.

Paraffin slides were deparaffinized and stained with a PAS staining kit (Sigma-Aldrich, 101646) according to the manufacturer’s protocol.

#### DAB stain.

Tissues were rehydrated using xylene and decreasing concentrations of ethanol. Antigen retrieval was performed in citrate buffer (pH 6.0) in a pressure cooker. Then endogenous peroxidase inhibition was performed using 3% H2O2/1XPBS followed by 3 washes in PBS. Tissues were blocked in CAS block (Thermo Fisher Scientific) and incubated overnight with primary antibodies diluted in CAS block (Cell Signaling, F4/80 Rabbit mAb 70076, 1:250). Slides were washed 3 times in PBST and incubated with the secondary antibody for DAB stain HRP horse anti-rabbit IgG Plus polymer KIT (ImmPRESS, MP-7801).

### COBAS analysis

Plasma samples were analyzed for alanine aminotransferase (ALT), aspartate aminotransferase (AST), alkaline phosphatase (ALP), triglycerides, cholesterol, LDL, HDL, and albumin using a Cobas c111 (Roche Diagnostics) automated clinical chemistry analyzer that was calibrated according to manufacturer guidelines.

### BCAA assay

Plasma-branched amino acids were measured using a commercially available kit (Abcam, ab83374). Plasma was taken following a 6-hour fast.

### Statistics

Results are presented as box plots with whiskers representing the highest and lowest values without outliers (1.5 IQR above or below the third or first quartile). Two-tailed Student’s *t* test, ANOVA, Mann-Whitney U test, and Kruskal-Wallis tests were used to assess differences between mean values between groups and post hoc tests were performed if *P* < 0.05. Bonferroni correction was used for multiple comparisons and Benjamini-Hochberg correction for statistical correction in RNA-Seq analysis.

### Study approval

The experiments were approved by the Hebrew University’s Institutional Animal Care and Use Committee.

## Author contributions

LH, RBHS, AR, and DBZ designed the experiments. LH, RBHS, YA, SA, MB, AH, HI, DK, YL, IS, DS, and DBZ performed experiments. LH, MB, RBHS, and DBZ analyzed the data. AR and DBZ conceived the study. DBZ acquired funding for the study. LH, RBHS, AR, and DBZ wrote the manuscript. Order of co–senior authors was agreed upon and based on the level of author involvement.

## Supplementary Material

Supplemental data

## Figures and Tables

**Table 2:**
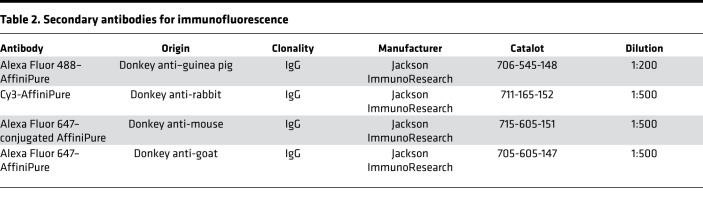
Secondary antibodies for immunofluorescence

**Table 1:**
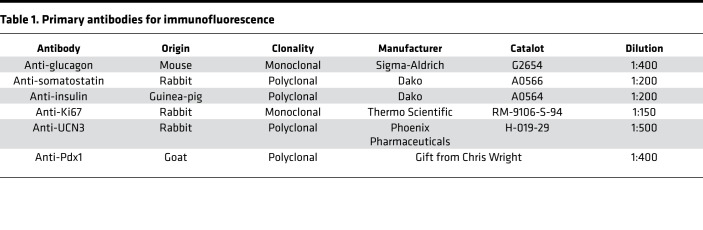
Primary antibodies for immunofluorescence

## Version 1 04/08/2022

Electronic publication
