# Supplementary material for: Maternal antagonism of Glp1 reverses the adverse outcomes of sleeve gastrectomy on mouse offspring
Source: JCI Insight. 2022 Apr 8;7(7):e156424. doi: 10.1172/jci.insight.156424 (PMC9057621; doi:10.1172/jci.insight.156424)
Supplement: Supplemental data [file jciinsight-7-156424-s260.pdf]

Maternal antagonism of Glp1 reverses the adverse outcomes of sleeve gastrectomy on mouse offspring

Supplementary information

Liron Hefetz[1,2], Rachel Ben-Haroush Schyr[1], Michael Bergel[1], Yhara Arad[1,2], Doron Kleiman[1], Hadar Israeli[1], Itia Samuel, Shira Azulai[1], Arnon Haran[1], Yovel Levy[1], Dana Sender[1], Amihai Rottenstreich[3,4,*], Danny Ben-Zvi[1*,#]

1. Dept of Developmental Biology and Cancer Research, Institute of Medical Research Israel-Canada, Hebrew University-Hadassah Medical School, Jerusalem, Israel
2. Department of Military Medicine and "Tzameret", Faculty of Medicine, Hebrew University of Jerusalem, Jerusalem, Israel, and Medical Corps, Israel Defense Forces, Israel
3. Department of Obstetrics and Gynecology, Hadassah-Hebrew University Medical Center, Jerusalem, Israel.
4. Faculty of Medicine, Hadassah-Hebrew University Medical Center, Jerusalem, Israel

*These authors are the senior authors of this paper

[#]Correspondence: Danny Ben-Zvi, danny.ben-zvi@mail.huji.ac.il, The Hebrew University of Jerusalem, Ein Kerem Campus, room 3038, Jerusalem, Israel. +972-5-472772406

Changes marked in yellow

Supplementary Figures

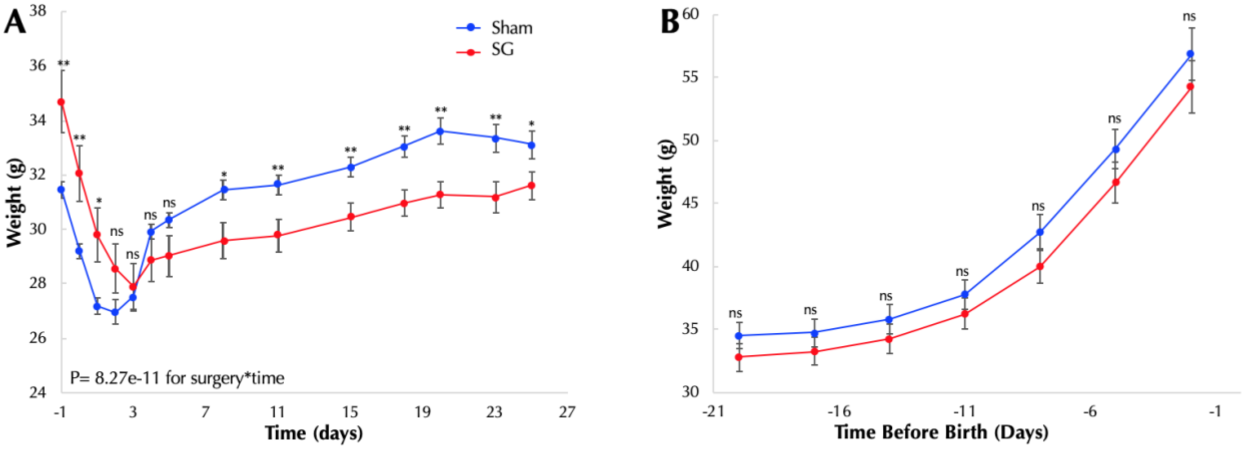

Figure S1: The effects of sleeve gastrectomy or sham surgery on female mice before and during pregnancy - related to Figure 1.

A-B. mice weight before pregnancy (A) and during pregnancy (B).

Blue: sham-operated mice, red: SG-operated mice. *,** p<0.05. 0.01 by 2-way continuous measure ANOVA. n=28,25 (Sham, SG accordingly) (A), 11,10 (B).

Figure S2. The effects of sleeve gastrectomy and sham surgery on the placenta and fetus. Related to Figure 2.

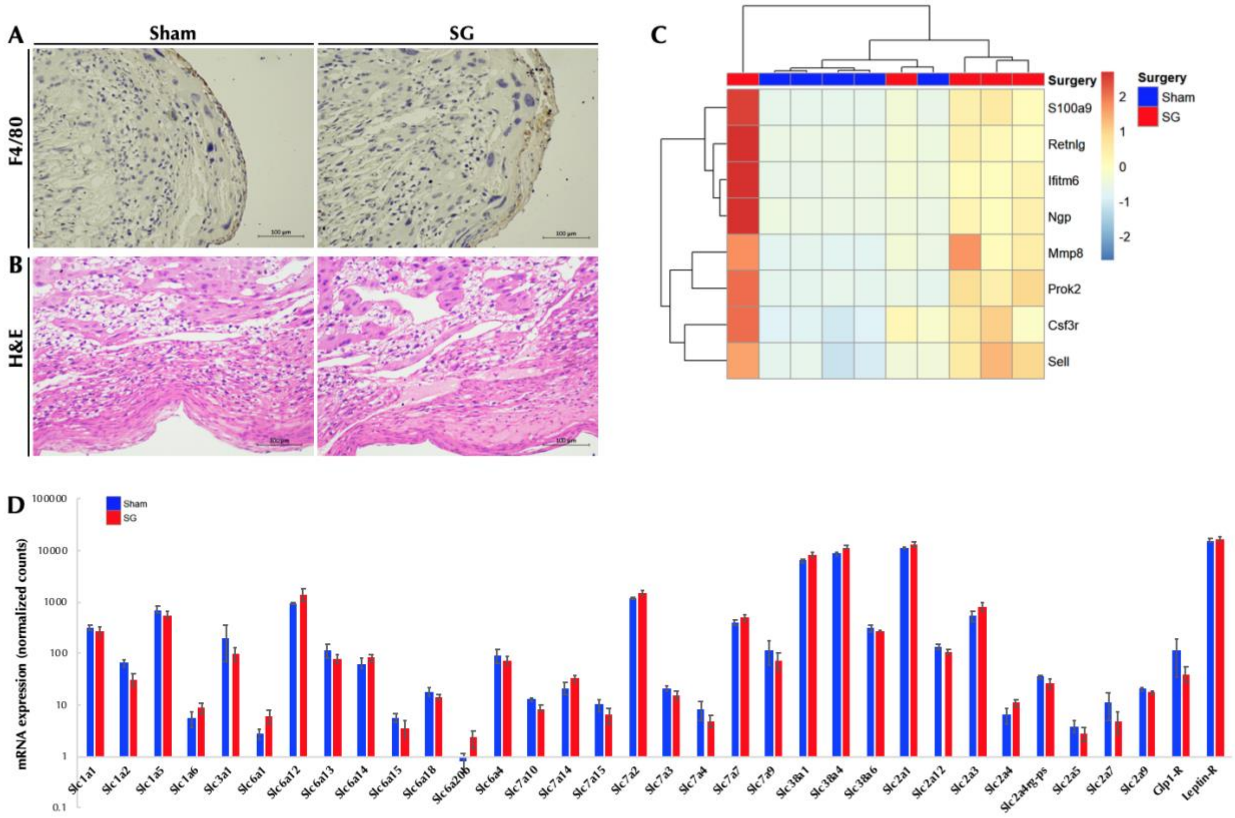

A. Representative immunostaining for F4/80, a marker for macrophages.

B. H&E staining of placentas

C. Differentially expressed genes in placentas from mice that underwent SG or Sham surgery. Shown are genes with adjusted p-value < 0.05.

D. Mean expression of transporters, the Glp1 receptor and leptin receptor in the placenta. Blue: placentas of sham-operated mice, red: SG-operated mice. All comparisons yielded non-significant difference by Student's t-test with FDR correction. n=5,5 placentas in C,D. a single placenta from each litter.

Figure S3: Offspring of sleeve gastrectomy operated mice are born small for gestational age. Related to Figure 3.

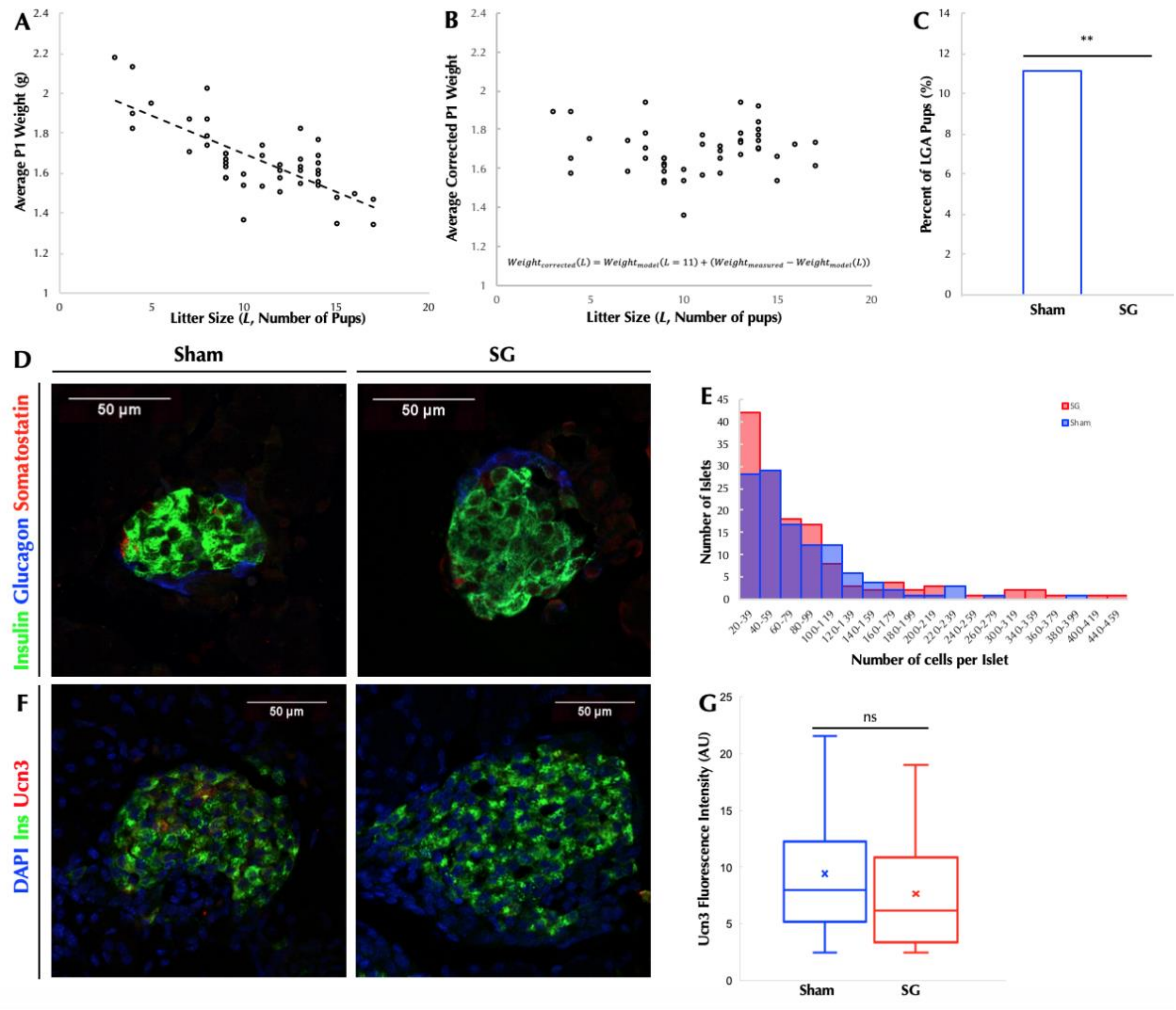

A. Pup weight as a function of litter size in sham operated mice. L stands for the number of pups. Dashed line denotes the linear trendline showing a decrease in average P1 weight as a function of increasing litter size.

B. Average corrected P1 weight as a function of litter size. There is no associated between average corrected weight and litter size. The model used to correct the weight is shown, with L the number of pups in litter of the specific mouse measured.

C. Percentage of LGA mice in sham and SG operated mice.

D. Images of P1 islets from offspring of SG and Sham-operated mice.

E. Distribution of islet size in P1 pups.

F-G. Representative images (F) and quantification (G) of UCN3 florescence in islets of P1 mice born to SG and Sham-operated mice.

Blue: offspring of sham-operated mice, red: SG-operated mice. ** $p<0.01$ by Chi-squared test (C).

n=137(17),117(14) islets(pups) (Sham, SG accordingly) (E), n=43(10),18(5) islets(pups) (F).

Figure S4. Offspring of sleeve gastrectomy operated mice remain smaller than offspring of sham operated mice until weaning. Related to Figure 4.

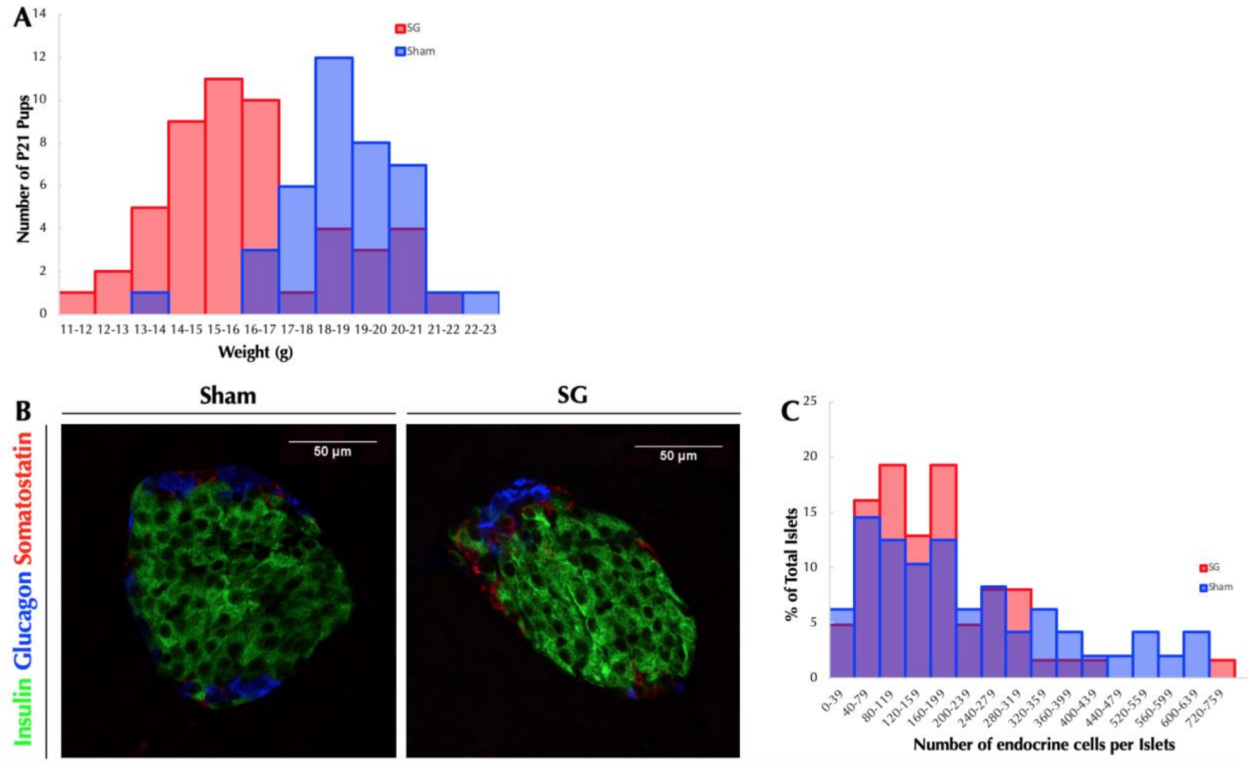

A. Distributions of weights of offspring of SG and sham-operated mice at P21.

B. Representative images of islets of offspring of SG and sham-operated mice at P21.

C. Distribution of islet size in offspring of SG and sham-operated mice at P21.

n=51,39 pups (SG, Sham) (A). n=155(51),141(39) islets(pups) (C).

Figure S5. Treatment with Exendin 9-39 normalizes the effects of SG on embryo size without affecting maternal glycemia. Related to Figure 5.

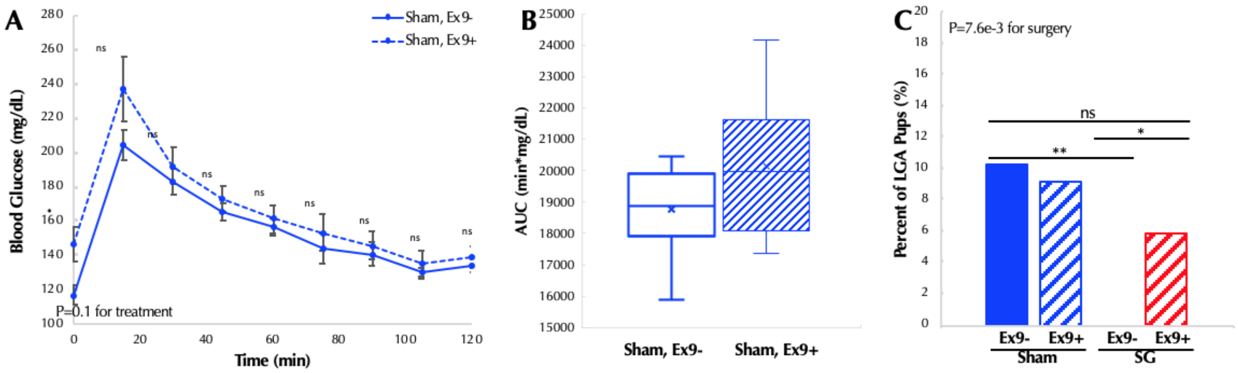

A-B. Oral glucose tolerance test (A) and area under the curve (B) of virgin unoperated mice treated or untreated with Exendin 9-39.

C. Percentage of LGA in pups born to Sham or SG-operated mice, treated or not treated with Exendin 9-39.

*,p<0.05 following 2-way ANOVA. n=9,9 (A-B).

Figure S6. The long-term effects of SG on female offspring and the effects of maternal treatment with Exendin 9-39.

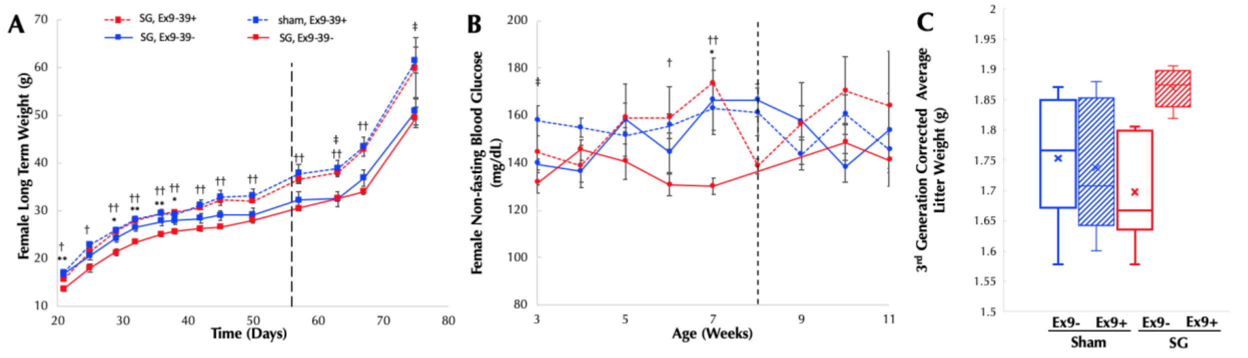

A-B. Weight (A) and non-fasting glucose levels of female offspring of Sham or SG-operated mice treated or untreated with Exendin 9-39 during pregnancy. Dashed black line denotes mating.

C. Adjusted mean weight of pups per litter at P1 in the four groups.

Blue: Female offspring of Sham-operated mice untreated with Exendin 9-39 during pregnancy (full) or treated with Exendin 9-39 (dashed); Red: Female offspring of Sham-operated mice untreated with Exendin 9-39 during pregnancy (full) or treated with Exendin 9-39 (dashed). *,**p<0.05, 0.01 by 2-Way ANOVA with Tukey HSD post-hoc test (C-E) or 3-way continuous measures ANOVA (A,B). n=4,9,7,3 (A,B), 8,8,7,6 (C) (Sham Ex9-, Sham Ex9+, SG Ex9-, SG Ex9+ accordingly).

*,** for the comparison between Sham-Ex9- and SG-Ex9-.

†, †† for the comparison between SG-Ex9- and SG-Ex9+.

‡, ‡‡ for the comparison between Sham-Ex9- and Sham-Ex9+.

Figure S7. The long-term effects of SG on male offspring are reversed by maternal treatment with Exendin 9-39. Related to Figure 6.

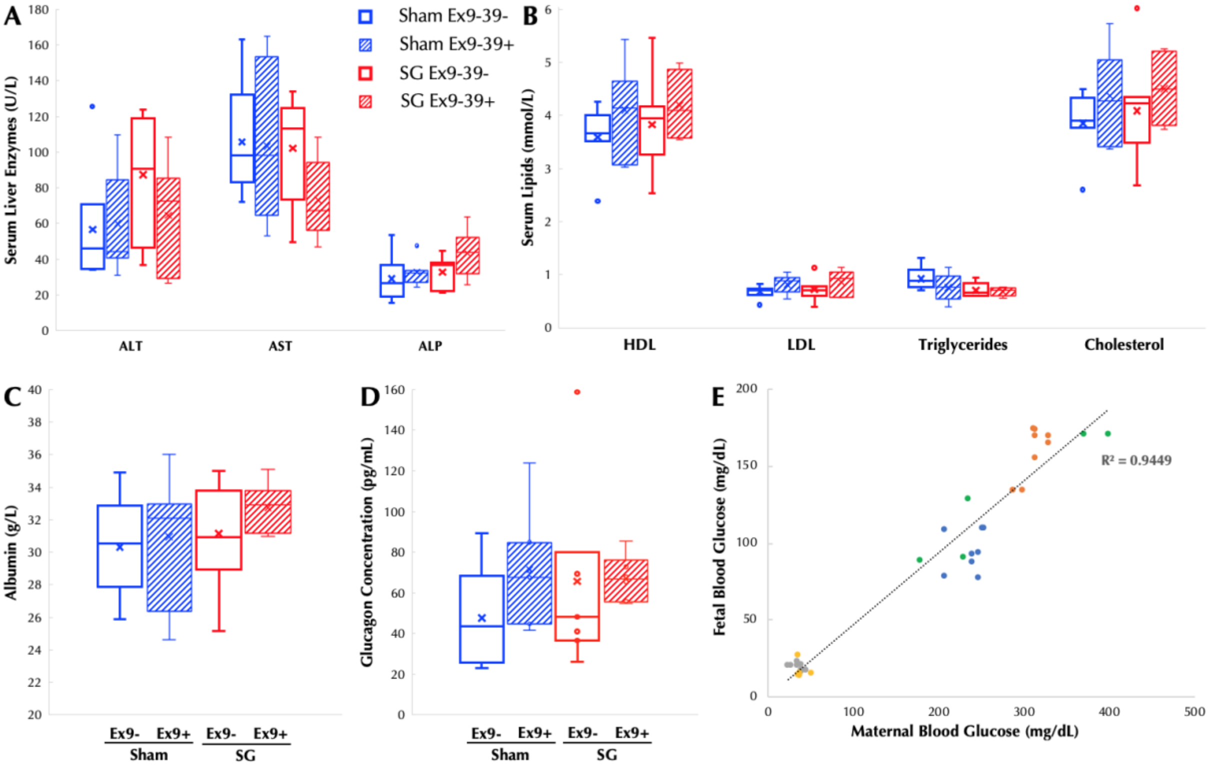

A-D. Plasma levels of hepatic enzymes (A), lipids (B), albumin (C) and glucagon (D) in the four experimental groups. n=7,7,7,6 in all panels Sham Ex9-, Sham Ex9+, SG Ex9-, SG Ex9+ accordingly.

E. Fetal blood glucose as a function of maternal blood glucose in sham operated mice. Each color represents a single offspring of the same dam.
